# Effect of Maternal Probiotic Supplementation During Breastfeeding on Postpartum Depression and Bilirubin Levels in Low-Birth-Weight (1000–2000 G) Infants: A Randomized Controlled Trial

**DOI:** 10.34172/hpp.45313

**Published:** 2026-08-21

**Authors:** Maryam Alikamali, Mahnaz Shahnazi, Mojgan Mirghafourvand, Manizheh Mostafa-Gharehbaghi, Aziz Homayouni-Rad, Zahra Fardiazar, Sakineh Mohammad-Alizadeh-Charandabi

**Affiliations:** ^1^Reproductive and Family Health Research Center, Kerman University of Medical Sciences, Kerman, Iran; ^2^Student Research Committee, Faculty of Nursing and Midwifery, Tabriz University of Medical Sciences, Tabriz, Iran; ^3^Department of Midwifery, Faculty of Nursing and Midwifery, Tabriz University of Medical Sciences, Tabriz, Iran; ^4^Social Determinants of Health Research Centre, Faculty of Nursing and Midwifery, Tabriz University of Medical Sciences, Tabriz, Iran; ^5^Pediatric Health Research Centre, Faculty of Medicine, Tabriz University of Medical Sciences, Tabriz, Iran; ^6^Department of Food Science and Technology, Faculty of Nutrition and Food Science, Tabriz University of Medical Sciences, Tabriz, Iran; ^7^Women’s Reproductive Health Research Centre, Faculty of Medicine, Tabriz University of Medical Sciences, Tabriz, Iran

**Keywords:** Breast feeding, Depression, Postpartum, Hyperbilirubinemia, Neonatal, Infant, Low birth weight, Probiotics

## Abstract

**Introduction:**

Evidence on the effects of maternal probiotic supplementation on postpartum mental health and neonatal outcomes in low birth weight infants remains limited. This study examined whether postpartum maternal oral supplementation with *Lactobacillus paracasei* 431 and *Bifidobacterium lactis* BB-12 (1×10⁹ CFU/day each) alleviates postpartum depression (PPD) symptoms and reduces total serum bilirubin (TSB) levels in infants weighing 1000–2000 g at birth (two primary outcomes).

**Methods:**

In this randomized, double-blind, placebo-controlled trial, women who had delivered within 48 hours and intended to breastfeed their infants received either probiotics or placebo for 42–45 days. Mothers—not infants—were the unit of randomization (stratified by twin status and birth-weight category). Postpartum depression was assessed using the Edinburgh Postnatal Depression Scale at baseline and at 42–45 days postpartum. TSB was assessed at baseline and on days 4 and 7 of the intervention. ANCOVA and repeated measures ANOVA, adjusted for baseline values and the stratification factors were used to compare the groups in terms of PPD score and TSB levels, respectively.

**Results:**

One hundred twenty-two mothers (138 infants, including 16 sets of twins) were enrolled and randomized into two groups. After one mother dropped per group, data from 60 mothers and 69 infants in each group were analyzed. Depression scores did not differ significantly between the probiotic and placebo groups (adjusted mean difference −1.6; 95% confidence interval [CI] −3.9 to 0.69). Similarly, infant TSB levels across days 4 and 7 of the intervention showed no significant differences (0.21; 95% CI: −0.31 to 0.73). One infant death and two cases of positive blood culture occurred in each group.

**Conclusion:**

The maternal probiotic supplementation during breastfeeding in mothers of infants weighing 1000–2000 g at birth did not significantly affect postpartum depression or infant TSB levels. Any potential benefit of higher probiotic doses or longer intervention periods remains speculative and should be weighed against neonatal safety considerations.

**Trial registration:**

IRCT20100414003706N42 (registered 22 November 2022, revised 4 February 2024; https://en.irct.ir/trial/65451).

## Introduction

 Postpartum depression (PPD) affects approximately 17% of women globally.^1^ A recent systematic review has indicated that preterm birth significantly increases the risk of PPD by approximately 29%.^2^ Despite the availability of psychotherapy and antidepressant treatments, many women with PPD either lack access to these interventions or choose not to pursue them.^3^

 Approximately 80% of premature infants develop clinical jaundice within the first week of life.^4^ Despite advancements in management, hyperbilirubinemia remains a major contributor to neonatal mortality and morbidity.^5^ Current management guidelines rely primarily on phototherapy; however, concerns persist regarding its potential adverse effects on premature infants.^6^ Therefore, exploring complementary approaches to these complications is warranted.

 Probiotics may alleviate symptoms of depression by modulating the gut microbiota and enhancing the production of neurotransmitters such as serotonin and gamma-aminobutyric acid in the brain. However, a systematic review found no statistically significant difference in depression levels between probiotic and control groups, indicating the need for further research into the potential effects of probiotics on maternal mental health.^7^

 Several studies have indicated no significantadverse effects of probiotics on breastfeeding women and their infants.^8,9^ However, rare cases of bacterial sepsis in neonates following direct probiotic administration have been reported, underscoring the need for cautionwhen administering probiotics directly topremature infants.^10^ Research has demonstrated the transmission of bacterial strains such as *Lactobacillus* and *Bifidobacterium*from mother to infant through breastfeeding,with a wide range of clinical benefits and a favorable safety profile.^11,12^ Probiotics can influence bilirubin concentration in the liver-intestinal circulation through various mechanisms, including inhibiting the degradation of conjugated bilirubin, promoting intestinal peristalsis, and enhancing defecation, ultimately leading to increased bilirubin excretion.^13^ Only a few small trials have assessed the effects of maternal probiotic supplementation on the morbidity of very-low-birth-weight (VLBW) infants.^14^ The trials demonstrated promising results.^15,16^

 Therefore, given the concerns regarding direct probiotic administration to preterm infants and the promising results of maternal administration, the present trial adopted a maternal route of probiotic administration. For safety, infants were prospectively monitored for adverse events, including clinical signs of neonatal sepsis, throughout the intervention period. Specifically, we aimed to assess the effects of oral probiotics containing *Lactobacillus paracasei* 431 and *Bifidobacterium lactis* BB-12, administered to breastfeeding mothers whose infantsweighed 1000–2000 g at birth, on two primary outcomes—one maternal (PPD) and one neonatal (total serum bilirubin [TSB] levels on days 4 and 7 of the intervention) —along with additional maternal and neonatal secondary outcomes.

## Methods

###  Study Design and Participants

 A detailed description of the study methods has been previously published in the study protocol.^17^ Therefore, we provide a concise explanation of the methods here.

 This study employed a randomized, double-blind, placebo-controlled trial design. Mothers—not infants—were the unit of randomization. It was conducted at Al-Zahra Educational Hospital in Tabriz, Iran, the leading referral center in northwest Iran for mothers delivering premature infants, especially those weighing under 1500 g, and also for such infants born in other hospitals.

 Participants included lactating women and their premature infant(s) who were born within the preceding 48 hours. In cases of twin births, both infants were included if they met the eligibility criteria. To be eligible for participation, both the mother and at least one of her infants had to meet the specified criteria. The initial target population was mothers of infants weighing 1000–1500 g; however, after approximately eight months, enrolment was slower than anticipated because of the limited number of eligible participants. To improve enrolment, the upper birth weight limit was raised to 2000 g through a protocol amendment approved by the Research Council, and the registration was updated in the IRCT on 4 February 2024, before any outcome analyses. Randomization was stratified by birth-weight category (1000–1500 g and 1501–2000 g) to control for potential confounding effects.

###  Inclusion Criteria

 Neonates were eligible if they had a birth weight between 1000 and 2000 g, were able to receive breast milk through any enteral feeding method (including gavage feeding, cup feeding, or direct breastfeeding), and were expected to remain hospitalized for at least 7 days after initiation of the intervention.

###  Exclusion Criteria

 Maternal exclusion criteria included regular consumption of probiotics in any form, a history of allergy to probiotics, immunodeficiency or known serious illnesses, and alcohol or substance abuse. Neonatal exclusion criteria comprised anomalies, instability (defined as a state of critical illness with unstable vital signs, such as significant hemodynamic or respiratory fluctuations, requiring intensive intervention), immunodeficiency, and a diagnosis of pathological jaundice at the time of enrolment, as diagnosed by a neonatologist.

###  Study Protocol

 The principal investigator (PI) first assessed potential participants for eligibility. For those deemed eligible, the PI obtained written informed consent from the participating women and, when possible, from their husbands or legal guardians before proceeding with baseline data collection and the intervention.

 Stratified blocked randomization was used, with randomly varying block sizes of 4 and 6 and a 1:1 allocation ratio. The allocation sequence was generated using the Random.org computer program, with the mother as the unit of randomization. Stratification was based on two factors: the number of infants (singleton or twins) and birth-weight category (1000–1500 g and 1501–2000 g). To maintain allocation concealment and blinding, participants were provided with identical, opaque, sequentially numbered bottles containing either probiotic or placebo capsules. The allocation sequence was generated and the bottles were prepared by a person who was not involved in participant recruitment, allocation, or data collection. Blinding was maintained for trial participants, care providers, outcome assessors, and data analysts. Unblinding was permitted at the request of the attending neonatologist; however, no cases of unblinding occurred.

 The probiotic strains *L. paracasei* 431 and *B. lactis* BB-12 were procured from Hansen Co., Denmark, and cornstarch was obtained from Glucose Co., Iran. Cornstarch served as an inert pharmaceutical excipient and filler for the probiotic strains. In the laboratory of the Pharmacy Department of Tabriz University of Medical Sciences, these strains were combined with cornstarch and encapsulated (each capsule containing 500 mg) to produce a probiotic supplement, while placebo capsules were filled solely with cornstarch. The capsules were identical in appearance and differed only in their probiotic ingredients. Each probiotic capsule contained at least 1 × 10⁹ colony-forming units (CFU) of each bacterial strain. Powder samples underwent microbiological culture testing to verify bacterial viability both before encapsulation and after completion of follow-up for all participants. These tests confirmed that the viability of both strains remained at ≥ 1 × 10⁹ CFU per capsule at baseline and at the end of the study.

 Participants received either the oral probiotic or the placebo capsules daily, preferably before or during a meal, starting from the day of recruitment and continuing for 42–45 days. They were instructed to store the capsules in a cool, dry place and to take them at least one hour before or after taking any other supplements and at least two hours apart from consuming oral antibiotics.

 To ensure accurate consumption, the capsules were administered directly to the mothers under the supervision of the PI while they were in the hospital. If an infant was discharged before completing the 42–45-day intervention period, the PI supplied the remaining capsules to the mother and conducted weekly follow-up telephone calls. Participants were required to document all capsule intake and any side effects in a diary. The actual amount of breast milk received by every infant was also monitored because it could influence probiotic transfer from mother to infant and, consequently, infant outcomes.

 Both intervention groups received standard care provided in the hospital and health centres. They were also encouraged to continue having kangaroo mother care. To minimize the influence of potential confounding factors on gut microbiota and bilirubin outcomes, feeding practices, and adherence to phototherapy protocols were prospectively monitored throughout the intervention and compared between the two intervention groups.

###  Outcome Measures

 The study had two primary outcomes: maternal depressive symptom score at six weeks postpartum and infant total serum bilirubin (TSB) levels measured on days 4 and 7 after initiation of the intervention. The six-week postpartum assessment aligns with the conventional timeframe for evaluating postpartum depression^18^ and corresponds to the end of our 42-45-day intervention, allowing for the examination of the immediate effect of the supplementation on maternal mental health. The measurement of infant TSB levels on days 4 and 7 was selected to monitor the peak and trend of jaundice in premature infants, a critical timeframe for this population. Moreover, this timing is consistent with our previous trial for comparative purposes.^15^ Baseline values of these variables were also assessed and incorporated into the corresponding analyses as adjustment variables.

 To assess maternal depressive symptoms, we utilized the validated Persian version of the Edinburgh Postnatal Depression Scale (EPDS).^19^ The scale was administered by the PI through an interview with each participant. In our study, Cronbach’s alpha was 0.78 at baseline and 0.87 at the six-week postpartum assessment, confirming internal consistency.

 Infant TSB levels were measured at the laboratory of Al-Zahra Educational Hospital. To ensure measurement reliability, the results of 10 samples were cross-verified between the hospital laboratoryand the city reference laboratory.

 Secondary maternal outcomes included the incidence of mastitis during the 42–45-day intervention period and anxiety scores at six weeks postpartum. Mastitis was diagnosed when a woman reported at least two breast symptoms and at least one flu-like symptom. We assessed general anxiety using the Persian-validated State Scale of Spielberger State-Trait Anxiety Inventory (SATI)^20^ at baseline, and postpartum-specific anxiety using the Postpartum Specific Anxiety Scale Research Short-Form (PSAS-RSF)^21^ at six weeks postpartum. In our study, Cronbach’s alpha was 0.86 for the SATI and 0.87 for the PSAS-RSF. It has been noted that using the specific scale to assess postpartum anxiety is preferable to general tools. However, such a scale cannot be used in the first postpartum days.^21^ The baseline general anxiety score was included as a covariate to account for pre-existing maternal anxiety when analysing postpartum-specific anxiety, while recognizing that the two instruments are not interchangeable.

 The main secondary infant outcomes included the total duration of phototherapy, weight gain over the 42–45-day period, length of infant hospitalization, and a composite outcome for the incidence of serious neonatal complications (including death, necrotizing enterocolitis, sepsis, bronchopulmonary dysplasia, retinopathy of prematurity, and intraventricular hemorrhage grade II or above) during the 42–45-day intervention.

 The PI conducted daily visits to the NICU to assess the outcomes by visiting the neonates, reviewing infant medical records, and consulting with the responsible neonatologists and nurses. The neonatologist from the research team assisted in ensuring accurate data collection within the NICU. All neonatologists adhered to the Queensland Maternity and Neonatal Clinical Guidelines^22^ for initiating and discontinuing phototherapy.

 At in-person visit on the 42^nd^ to 45th day, the PI measured the weights of the mothers and infants, reviewed ophthalmologist findings for retinopathy in infants, and inquired about blood transfusions, rehospitalisations, feeding types in the preceding 24 hours, and the overall well-being of the infants. Additionally, any unused capsules and completed diaries were collected.

 Potential side effects of probiotics, including sepsis, vomiting, loose stools, abdominal distension, and bloating, were listed and documented in a daily diary. The diary also included one open-ended item to record any additional side events. Over the 42–45-day intervention period, the diary was completed prospectively each day by PI, who reviewed infant medical records and queried the responsible neonatologists, nurses, other health personnel on the ward, and the mother. When necessary, the research collaborator and neonatologist (MMG, a highly experienced faculty member) was consulted to resolve any diagnostic uncertainties.

###  Sample Size 

 The sample size was calculated using a t-test for the difference between two independent means in G*Power software, based on the primary outcomes. For both outcomes, we used a two-sided significance level of 0.05 (with no adjustment of alpha level for the two primary endpoints) and a 1:1 allocation ratio.

 For the PPD outcome, based on a previous study conducted in Iran (mean = 7.18, SD = 3.99)^23^ and aiming to detect a clinically significant 30% reduction attributed to the intervention with 80% power, a required sample size of 61 participants per group (122 mothers in total) was determined after adding a 10% allowance for potential attrition.

 For the infant TSB outcome, based on data from a previous study in the same setting (mean = 6.0 mg/dL, SD = 2.3)^15^ and aiming to detect a clinically significant 25% reduction with 90% power, the required sample size was calculated as 51 participants per group. Therefore, the larger sample size of 61 mothers per group was selected as the final target sample size to provide adequate power for both outcomes, allowing for 10% attrition.

###  Statistical Analysis

 After data accuracy and completeness were verified, a modified intention-to-treat (mITT) analysis was employed, excluding one mother from each group who was lost to follow-up. For postpartum depressive symptom scores, postpartum anxiety scores, and infant weight gain, analysis of covariance (ANCOVA); and for post-intervention TSB levels, repeated measures ANOVA were used to compare intervention groups while adjusting for the corresponding baseline score and the prespecified stratification factors used in randomization. The group-by-time interaction was used to assess whether the change in TSB over post-intervention time differed between the intervention groups. The tests were performed after verifying the assumptions, including linearity of the relation between baseline and post-intervention scores at each intervention group, homogeneity of regression slopes, normality of residuals, and homogeneity of variance. Independent samples t, Mann-Whitney, chi-squared and Fisher’s exact tests were employed to compare the other secondary/exploratory outcomes between intervention groups.

 As an additional exploratory analysis, subgroup analyses were conducted according to birth weight category. This decision was made at the time of the population extension. Results for the VLBW subgroup (1000–1500 g) were presented in the main tables, reflecting the initially intended population, while results for the other birth-weight subgroup were presented in the supplementary tables.

 A two-sided *P* value < 0.05 was considered statistically significant. All statistical analyses were performed using IBM SPSS Statistics for Windows, version 24.0 (IBM Corp., Armonk, NY, USA).

## Results

 Between December 30, 2022, and November 25, 2023, a total of 122 women and their 138 infants (including 16 sets of twins) were enrolled in the study and randomly allocated to the two intervention groups. The follow-up phase was completed on January 10, 2024. One mother from each group was lost to follow-up following the death of her infant. Therefore, data from 120 mothers and 138 infants were analyzed (Figure 1). The observed attrition rate was 1.6%, which was lower than the 10% anticipated in the sample-size calculation.

**Figure 1:**
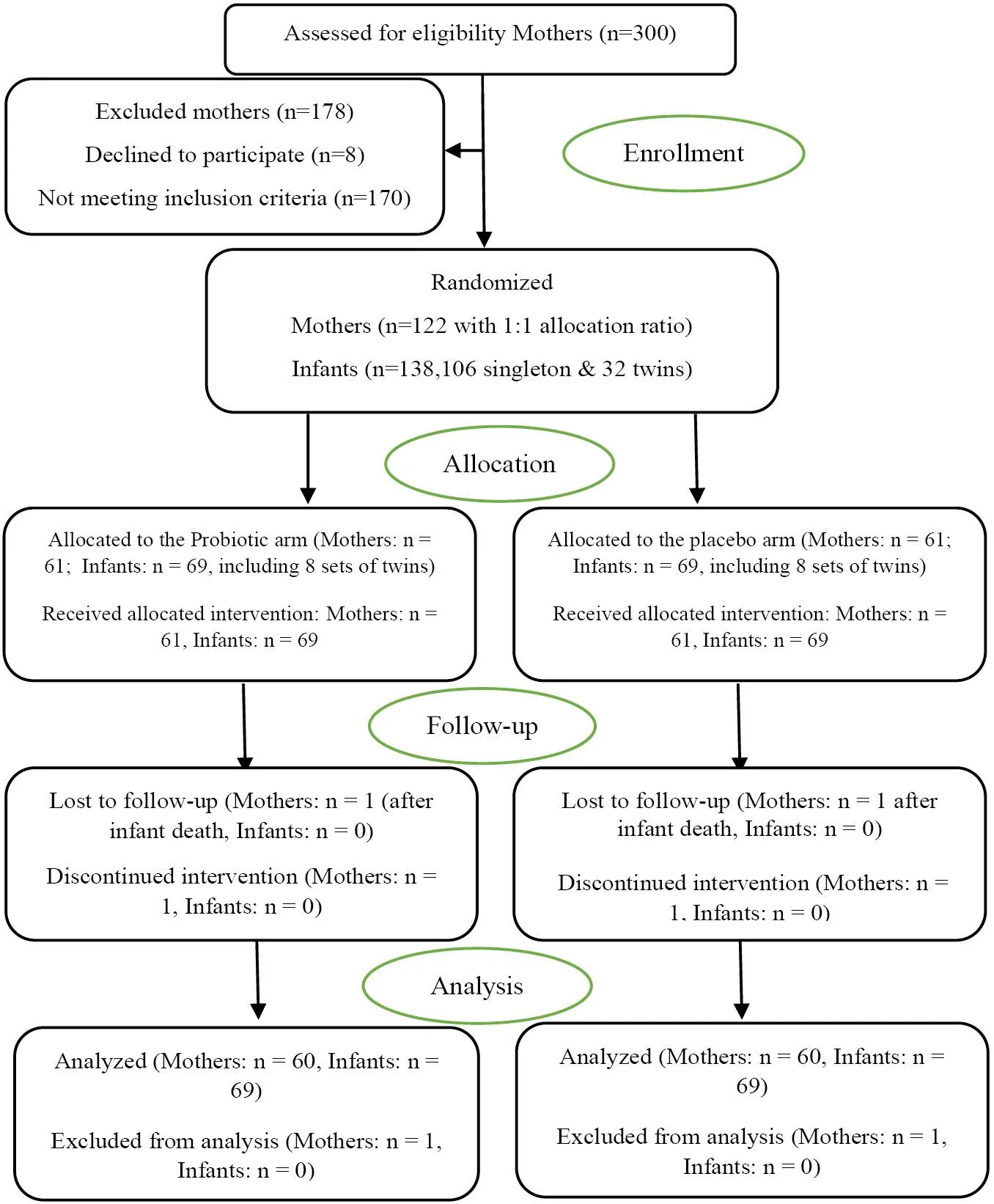
Flowchart of the trial process

 Evaluation of the returned capsule counts and daily diary records indicated complete adherence to the assigned intervention in both intervention groups, with no missed doses reported. However, most infants experienced varying periods without their mothers’ milk. In the probiotic group, 3 infants received no maternal breast milk for ≥ 11 days, 16 infants for 5–10 days, and 35 infants for 1–4 days. In the placebo group, the corresponding numbers were 2, 12 and 47 infants, respectively. The main reason for the temporary discontinuation was the need to withhold oral feeding during blood transfusions, which occurred in 26 infants in the probiotic group and 24 infants in the placebo group.

 Baseline characteristics of mothers and their infants showed no clinically relevant imbalances between the groups (Table 1, Table S1). Among the 138 infants, 78 (56%) had a birth weight of ≤ 1500 g (VLBW), representing 68 mothers.

**Table 1:** Baseline characteristics of mothers and infants by intervention group

	**All neonates**		**Very-low-birth-weight neonates**	
	**Probiotic**	**Placebo**	**Probiotic**	**Placebo**
**Maternal Characteristics**	**(n=61)**	**(n=61)**	**(n=36)**	**(n=32)**
Age (years)	31.4 [7.8]	31.3 [6.8]	31.1 [7.6]	30.4 [6.7]
Primipara	32 (52.5)	24 (39.3)	20 (55.6)	13 (40.6)
Education (less than 12 years)	15 (24.6)	22 (36.1)	7 (19.5)	10 (31.3)
Delivery with cesarean section	54 (88.5)	54 (88.5)	31 (86.1)	29 (90.6)
Important maternal complications^*^	21 (34.4)	28 (45.9)	9 (25.0)	15 (46.9)
Chronic hypertension	2 (3.3)	2 (3.3)	0 (0.0)	2 (6.3)
Preeclampsia	18 (29.5)	25 (41.0)	9 (25.0)	15 (46.9)
Diabetes	5 (8.2)	5 (8.2)	1 (2.8)	1 (3.1)
**Infant characteristics**^†^	**(n=69)**	**(n=69)**	**(n=41)**	**(n=37)**
Gestational age (weeks)	31.4 [1.9]	31.6 [2.3]	30.7 [1.8]	30.3 [2.2]
Birth weight (g)	1486 [294]	1478 [274]	1287 [168]	1273 [162]
Infant sex (boy)	34 (49.3)	32 (46.4)	18 (43.9)	19 (51.4)
Apgar score 5th min < 7	16 (23.2)	21 (30.4)	13 (31.7)	17 (45.9)
Received antenatal steroids	67 (97.1)	68 (98.6)	41 (100)	36 (96.9)
Meconium aspiration	2 (2.9)	1 (1.4)	2 (4.9)	1 (2.7)

Values are presented as number (%) or Mean [standard deviation] * Important maternal complications included chronic hypertension, preeclampsia, and diabetes mellitus ^†^Mothers were the unit of randomization, infant characteristics are presented at the infant level (69 infants including 8 twin sets per group)

 Overall, there were no statistically significant differences between the groups in either primary outcome: the depression symptom score at 42–45 days postpartum (adjusted mean difference −1.6, 95% CI −3.9 to 0.69; Table 2) and infant total serum bilirubin levels on days 4 and 7 of the intervention (adjusted mean difference 0.21, 95% CI −0.31 to 0.73; Table 3).

**Table 2:** Comparison of the intervention groups on the maternal outcomes

	**Mother of all neonates**			**Mother of very-low-birth-weight neonates**		
**Maternal outcomes**	Probiotic(n=61*)	Placebo(n=61*)	**AMD**^†^ ** (95% CI), P**	Probiotic(n=36*)	**Placebo(n=32)**	**AMD**^†^ ** (95% CI), ** ***P***
**Depression**^a^ ** (Primary outcome), (0-30)**						
Baseline	7.9 [5.1]	8.8 [4.8]		8.3 [5.3]	9.8 [5.2]	
42-45 postpartum days	5.2 [5.1]	6.0 [5.3]	-1.6 (-3.9 to 0.7), 0.168	7.6 [5.0]	7.6 (5.4]	-0.8 (-3.9 to 2.3), 0.61
**Anxiety**^b^ ** (Secondary outcome)**						
Baseline (20-80)	38.5 [10.7]	39.1 [9.8]		39.2 [11.3]	39.9 (11.4]	
42-45 postpartum days (16-64)	25.6 [7.0]	27.0 [6.9]	-3.5 (-7.0 to -0.03), 0.048^‡^	28.4 [7.4]	27.7 [7.7]	-2.2 (-7.1 to 2.6), 0.36^‡^

Values are presented as mean [standard deviation] unless otherwise indicated a Using the Edinburgh Postnatal Depression Scale (EPDS); higher scores indicate more severe symptoms b Using the State scale of the State-Trait Anxiety Inventory (STAI) to assess baseline anxiety, and the Postpartum Specific Anxiety Scale–Research Short-Form (PSAS-IR-RSF) to assess anxiety at 42-45 days postpartum; higher scores indicate more severe symptoms. Because anxiety was measured with different validated tools at the two time points, scores are not directly comparable across time * At 42-45 postpartum days: in the total group, n = 60 in each group; in the VLBW subgroup, n = 35 in the probiotic group due to loss to follow-up after infant death ^†^AMD: adjusted mean difference (probiotic minus placebo) at 42-45 days postpartum, estimated using ANCOVA and adjusted for stratification factors (twin status and birth-weight category) and the baseline values (baseline EPDS score for depression and baseline STAI score for anxiety) ^‡^Although the between-group difference was statistically significant in the total group and non-significant in the subgroup, interaction effect of intervention group × birth-weight group was not statistically significant (*P* = 0.45)

**Table 3:** Comparison of neonatal outcomes between intervention groups

**Neonatal outcomes**	**All neonates**			**Very-low-birth-weight neonates**^a^		
	**Probiotic** **(n=69)**	**Placebo** **(n=69)**	**AMD (95% CI)/ ** ***P*** **-value**	**Probiotic** **(n=41)**	**Placebo** **(n=37)**	**AMD (95% CI)/ ** ***P*** **-value**
**Total serum bilirubin (mg/dL), Primary outcome**						
Baseline	8.0 [1.8]	8.1 [2.2]		8.0 [1.9]	7.8 [2.1]	
4th day of intervention	5.9 [1.5]	5.8 [1.6]	0.21 (-0.31 to 0.73), 0.42^b^	5.8 [1.5]	5.7 [1.5]	0.46 (-0.15 to 1.07), 0.14^b^
7th day of intervention	4.4 [1.5]	4.2 [1.5]		4.2 [1.5]	3.9 [1.1]	
**Secondary outcomes**						
Phototherapy duration* (days, since birth)	4 [3, 6]	4 [3, 6]	0.80^c^	5 [4, 6]	5 [3.5, 6]	0.48^c^
Weight gain (g) during 42-45th days*	783 [410]	826 [402]	81 (-73 to 235),0.30^d^	695 [328]	671 [290]	57 (-93 to 207), 0.5^d^
Infant nutrition						
Duration of total parenteral nutrition^†^ (days, since birth)	1.5 [0, 12.8]	0 [0, 14]	0.88^c^	11.5 [4.3, 17.8]	14 [7.5, 20.5]	0.40^c^
Time to achieve full enteral feeding^†^ (days, since birth)	16 [10, 24.8]	15 [10, 20]	0.41^c^	22 [15, 31.8]	19 [16, 29.5]	0.85^c^
Breast milk received during the initial 7 days of the intervention (mL)	232 [96, 352]	200 [116, 372]	0.41^c^	128 [80, 248]	152 [92, 234]	0.62^c^
Length of hospital stay^†^ (days, since birth)	24 [12, 35]	18 [11, 29.5]	0.17^c^	32 [23.3, 43]	29 [20.5, 53]	0.70^c^
Duration of continuous positive airway pressure (days, since birth)	3 [1.5, 5]	2 [1, 5]	0.69^c^	3 [2, 6]	4 [2, 7]	0.49^c^
	**RR (95% CI)/ ** ***P*** **-value**					**RR (95% CI)/ ** ***P*** **-value**
Any serious complications^*‡^	13 (18.8)	18 (26.1)	0.82 (0.57 to 1.18)^e^	12 (29.3)	16 (43.2)	0.73 (0.5 to 1.1)^e^
Death	1 (1.4)	1 (1.4)		1 (1.4)	0 (0)	
Necrotizing enterocolitis	0 (0)	1 (1.4)		0 (0)	1 (2.7)	
Positive blood culture	2 (2.9)	2 (2.9)		2 (4.9)	2 (5.4)	
Bronchopulmonary dysplasia	11 (16.2)	13 (18.8)		10 (25.0)	13 (35.1)	
Retinopathy of prematurity	0 (0)	2 (2.9)		0 (0)	1 (2.7)	
Intraventricular hemorrhage (grade Ⅱ-IV)	0 (0)	2 (2.9)		0 (0)	2 (5.4)	
Received blood transfusion	26 (38.2)	24 (35.3)	1.07 (0.76 to 1.50)^e^	20 (50.0)	22 (59.5)	0.81 (0.49 to 1.3)^e^
Received phototherapy	69 (100)	67 (97.1)	0.50^f^	41 (100)	36 (97.3)	0.48^f^
Feeding intolerance on the 7th day of intervention	8 (11.6)	9 (13.0)	0.93 (0.55 to 1.59)^e^	8 (19.5)	7 (18.9)	1.0 (0.6 to 1.7)^e^
Nutrition with only breast milk at the discharge^†^	48 (70.6)	48 (69.6)	1.0 (0.7 to 1.4)^e^	26 (65.0)	24 (64.9)	1.0 (0.6 to 1.6)^e^
Hospital re-admission	3 (4.4)	3 (4.4)	> 0.99^f^	2 (5.0)	1 (2.7)	> 0.99^f^

Values are presented as number (%), mean [SD], or median [Q1, Q3], unless otherwise indicated *Main secondary outcomes ^†^In the probiotic group, 68 in the all neonates, and 40 in the VLBW subgroup due to no assessment possibility after infant death ^‡^Serious complications (composite outcome) included: death, necrotizing enterocolitis, positive blood culture, bronchopulmonary dysplasia, intraventricular hemorrhage (grade two or over), or retinopathy of prematurity ^a^ Subgroup results are exploratory and underpowered ^b^Repeated Measures ANOVA was performed, with the values at day 4 and day 7 as dependent variables, the baseline value as covariate, and the intervention group and stratification factors (twin status and birth-weight subgroup) as between-subject factor, with Sidak adjustment for multiple comparisons; Neither the group-by-time interaction nor the post-intervention time-specific effects were not significant (*P* = 0.66 and 0.72, respectively, for the all neonate group; and 0.24 and 0.86 for the VLBW subgroup) ^c^Mann-Whitney test ^d^ANCOVA adjusted for birth weight and stratification factors (twin status and birth-weight category) ^e^Chi-square test ^f^Fisher’s Exact test

 For the maternal secondary outcomes, the mean anxiety score was lower in the probiotics group than the control group at 42–45 days of intervention (*P* = 0.048; Table 2). No cases of mastitis occurred among the women and no women reported any side effects.

 For the main neonatal secondary outcomes, the composite of serious neonatal complications (including death, necrotizing enterocolitis, Sepsis, bronchopulmonary dysplasia, retinopathy of prematurity, and intraventricular hemorrhage) was less frequent in the probiotic group (19%) than the control group (26%), although the difference was not statistically significant (*P* = 0.30). There was one case of infant mortality and two cases of culture-positive sepsis in each group; one infant mortality and all of the sepsis occurred among VLBW infants (Table 3). Furthermore, no significant differences were found in the frequency of phototherapy (*P* = 0.50) or nutrition with only breast milk at discharge (*P* = 0.82) (Table 3).

 In exploratory subgroup analyses according to birth-weight category, there was no statistically significant differences between the intervention groups in mean anxiety score among mothers whose infants weighed ≤ 1500 g at birth (Table 2). Among mothers whose infants weighed 1501–2000 g at birth, the mean anxiety score was significantly lower in probiotic group than in the control group (Table S2). However, the interaction between intervention group and birth-weight category was not statistically significant (*P* = 0.45), indicating that there was no statistically significant evidence of a differential intervention effect across birth-weight categories. No other statistically significant differences were found between the intervention groups for the remaining outcomes in either birth-weight subgroup (Tables 2, 3, S2, and S3).

## Discussion

 The probiotic supplementation did not result in statistically significant between-group differences in either of the primary outcomes: postpartum depressive symptoms or infant TSB levels. Postpartum anxiety symptoms were significantly lower in the probiotic group than the placebo group in the overall study population and within the subgroup of mothers of infants weighing 1501–2000 g at birth. No statistically significant between-group differences were observed for the other secondary maternal or neonatal outcomes assessed.

 To our knowledge, there are currently no trials assessing the effects of postpartum probiotic supplementation on maternal mental health, and existing trials^24-27^ examining the effects of antenatal probiotic supplementation on antenatal and postnatal mental health have yielded inconclusive results. A recent systematic review indicated a significant reduction in postpartum depression scores associated with probiotic use; however, it found no significant effects on antenatal depression, antenatal anxiety, or postnatal anxiety, with considerable heterogeneity across studies.^28^ In almost all the included trials,^25-27^ probiotic supplementation commenced during the first trimester of pregnancy.

 In our study, the statistically significant lower postpartum anxiety score observed in the probiotics group compared with placebo group in the overall study population should be interpreted cautiously because anxiety was one of several secondary outcomes and the results were not adjusted for multiplicity. Therefore, this finding should be considered as hypothesis-generating rather than definitive evidence of efficacy. The lack of a statistically significant differences among mothers of infants weighing ≤ 1500 g at birth does not demonstrate a different intervention effect in this subgroup, because the intervention-by-birth-weight interaction was not statistically significant. The finding may reflect the limited sample size and insufficient power for the subgroup analyses.

 The lack of a significant effect of probiotics on infant TSB levels observed on day 4 in the current study is consistent with the results of our previous trial,^15^ which was conducted in the same setting. However, in the previous trial, we observed a significant reduction in TSB levels on day 7 of the intervention. Whereas the previous trial employed a single-strain supplement containing *L. paracasei* at a higher daily dosage of 1.5 × 10⁹ CFU, the current study used a multi-strain formulation containing *L. paracasei* at a lower dosage of 1 × 10⁹ CFU. Given that feeding practices and NICU protocols were generally similar across the two studies, differences in strain composition and dosage may partly explain the observed differences between the two trials.

 To date, we have not identified any other studies examining the effects of maternal postnatal probiotic administration on infant TSB levels. As noted by the authors of a recent review, limited and low-certainty evidence suggests that direct probiotic supplementation in neonates may reduce the duration of phototherapy, as well as in TSB levels at 96 hours and seven days following probiotic treatment, but not TSB levels at 72 hours or at shorter post-intervention periods.^29^ The discordant findings between our study and those of the review regarding TSB levels at seven days post-intervention may partly reflect the indirect nature of probiotic administration in our trial. Recent studies suggest that direct administration of probiotics to preterm infants may be more effective than indirect maternal administration for enhancing infant gut microbiota.^30,31^

 In our study, incomplete exposure to maternal breast milk among the majority of the infants, together with the relatively low volume of breast milk received, particularly by VLBW infants, may have limited the opportunity for maternal probiotic-derived bacterial transmission during the early postnatal period. Therefore, future studies should consider evaluating maternal probiotic supplementation in populations of infants who receive predominantly or exclusively their own mothers’ milk, as this may provide greater opportunity for probiotic transfer.

 Reviews have indicated that direct probiotic supplementation may improve certain outcomes in premature infants, including time to reach full feeding, length of hospital stay, and length of food intolerance.^32,33^ The differences between our findings and those reported in these reviews may partly reflect the indirect administration of probiotics to the infants in our study and the incomplete exposure to maternal breast milk among our participants.

 Although the incidence of serious neonatal complications was numerically lower in the probiotic group than in the control group, this difference was not statistically significant. Given the limited sample size and the low frequency of the serious neonatal events, this finding should be interpreted cautiously and may be considered hypothesis-generating for future adequately powered studies.

 The lack of intervention effects on the short-term outcomes examined in this trial may also be related to the high prevalence of antibiotic exposure among mothers and infants, which may have affected probiotic viability and maternal-to-infant microbiota transfer. Individual-level antibiotic exposure data were not systematically collected in this trial, precluding quantitative comparisons of antibiotic exposure between the intervention groups. However, antibiotic exposure was uniformly high across both intervention groups because of routine hospital protocols. Among mothers, 88.5% underwent cesarean delivery and received standard postpartum antibiotic regimens (2 g of cefazolin every 6 hours for the first 24 hours, followed by 400 mg of cefixime daily for one week), while the remaining women received at least 400 mg of cefixime daily for one week. All admitted infants received systemic antibiotics during the NICU stay, most commonly ampicillin, gentamicin, and tazobactam for an average duration of two weeks; broad-spectrum agents such as vancomycin and meropenem were administered to critically ill neonates. A recently published systematic review no clear evidence that probiotic supplementation prevents antibiotic-induced dysbiosis.^34^ However, another study demonstrated that concurrent probiotic administration during antibiotic treatment may have more favorable effects on gut microbiota recovery than probiotic administration initiated after antibiotic therapy has ended.^35^

###  Strengths and Limitations

 To the best of our knowledge, this trial is the first to investigate the effectiveness of combined probiotic supplementation with *Lactobacillus* and *Bifidobacterium* in breastfeeding mothers on postpartum depressive symptoms and TSB levels in LBW infants. The study also had several methodological strengths, including randomization; allocation concealment; blinding of participants, all clinical investigators, data collectors, and data analysts; near-complete follow-up (only one mother dropout per group); high adherence to the intervention; and analysis and reporting of all outcomes according to the prespecified protocol.

 Nevertheless, several limitations should be considered when interpreting the findings. First, the study population was expanded during recruitment by increasing the upper birth-weight eligibility criterion to facilitate recruitment. This change may have increased the clinical heterogeneity of the study population. Second, the potential non-independence of outcomes within twin pairs was not formally accounted for in the statistical analyses. Although twin pairs represented only a small proportion of the study population, this should be considered when interpreting the infant-level findings. Third, the extensive antibiotic exposure among mothers and infants may have influenced the study findings, and the incomplete breast-milk exposure among infants may have affected the infant outcomes. Fourth, because of the limited sample size, some key outcomes, such as serious infant complications, were designated as secondary outcomes, yielding inconclusive results. In addition, the intervention and follow-up duration were relatively short. Furthermore, no adjustment for potential multiplicity arising from the assessment of two primary outcomes was performed. The results of the multiple secondary outcome analyses and exploratory subgroup analyses should only be regarded as hypothesis-generating. Finally, due to resource constraints, we were unable to assess bacterial colonization in biological samples obtained from mothers and infants.

 The statistically non-significant trend toward a lower incidence of serious neonatal complications in the probiotic group should not be interpreted as evidence of an intervention effect. However, the observed direction of effect may warrant further investigation in larger-scale studies in which such outcomes are prespecified as primary endpoints. Future studies should also employ longer intervention and follow-up periods to assess potential long-term effects on maternal and infant health, use appropriate statistical methods to account for potential non-independence of twin outcomes and to mitigate or account for multiplicity, and include the analysis of biological samples, such as infant stool, to assess bacterial colonization.

## Conclusion

 Administration of probiotics containing *Lactobacillus paracasei* and *Bifidobacterium animalis*, each at a concentration of 1 × 10^9^ CFU/day, to breastfeeding women with infants weighing 1000- 2000 g at birth did not significantly affect postpartum depressive symptoms or infant total TSB levels. The statistically significant reduction in the postpartum anxiety symptoms should be interpreted cautiously because this finding was not based on a primary outcome analysis. Further research with longer intervention and follow-up periods is warranted to determine the potential long-term effects of maternal probiotic supplementation in this vulnerable population. Given the limitations associated with the indirect administration route and the low volume of maternal breast milk intake among these infants, future studies could cautiously investigate whether higher probiotic doses or longer intervention periods provide additional benefits. However, any potential benefit of higher probiotic doses or prolonged intervention remain speculative and should be weighed against maternal and neonatal safety considerations.

## Competing Interests

 The authors declare that they have no competing interests.

## Data Availability Statement

 The datasets used and/or analyzed during the current study are available from the corresponding author on reasonable request.

## Ethical Approval

 The privacy rights of all participants have been observed. Written informed consent was obtained from all patients participating in this study. Participant recruitment was started after ethical approval from the Clinical Research Ethics Committee of Tabriz University of Medical Sciences andtrial registration in Iranian Clinical Trials Registry (code: IRCT20100414003706N42 Registration date: 2022/11/22, revised on 2024/2/4 (https://en.irct.ir/trial/65451).

 Supplementary information: Supplementary Tables 1–3, presenting baseline characteristics and detailed maternal and neonatal outcomes for the 1501–2000 g birth-weight infants subgroup, are available as supplementary material.

## Supplementary Files

Supplmentary file contains table S1, S2 and S3
