# Supplementary material for: Effect of Maternal Probiotic Supplementation During Breastfeeding on Postpartum Depression and Bilirubin Levels in Low-Birth-Weight (1000–2000 G) Infants: A Randomized Controlled Trial
Source: Health Promot Perspect. 2026 Aug 21;16(2):272–81. doi: 10.34172/hpp.45313 (PMC13723429; doi:10.34172/hpp.45313)
Supplement: Supplementary file 1 [file hpp-16-272-s001.pdf]

Alikamali et al, Health Promotion Perspectives 2026;16(2):272-S1
doi:10.34172/hpp.45313
https://hpp.tbzmed.ac.ir

Supplementary Table 1. Baseline characteristics of the intervention groups in the subgroup of mothers whose infants weighed 1501–2000 g at birth

**Maternal Characteristics**	**Probiotic (n = 25)**	**Placebo (n = 29)**
Age (years)	31.8 [8.2]	32.4 [6.9]
Primipara	12 (48.0)	11 (37.9)
Education (less than 12 years)	8 (32.0)	12 (41.4)
Delivery with cesarean section	23 (92.0)	25 (86.2)
Important maternal complications[a]	12 (48.0)	13 (44.8)
Chronic hypertension	2 (8.0)	0 (0)
Preeclampsia	9 (36.0)	10 (34.5)
Diabetes	4 (16.0)	4 (13.8)
**Infant characteristics**	**(n = 28)**	**(n = 32)**
Gestational age (weeks)	32.5 [1.5]	33.0 [1.4]
Birth weight (g)	1776 [167]	1715 [161]
Infant sex (boy)	16 (57.1)	13 (40.6)
Apgar score 5th min < 7	3 (10.7)	4 (12.5)
Received antenatal steroids	26 (92.9)	32 (100)
Meconium aspiration	0 (0)	0 (0)

Values are number (%) or mean [SD] unless otherwise indicated

[a] Important maternal complications included chronic hypertension, preeclampsia, and diabetes mellitus.

Supplementary Table 2. Comparison of maternal outcomes between the intervention groups among women whose infants weighed 1501-2000 g at birth

Mother of low-birth-weight neonates			
**Maternal outcomes**	**Probiotic (n = 25)**	**Placebo (n = 29*)**	**AMD (95% CI), P[a]**
**Depression score**, (0-30)[†]			
Baseline	7.4 [4.8]	7.7 [4.2]	
42- 45 postpartum days	1.9 [2.8]	4.2 [4.6]	-2.4 (-5.7 to 0.88), 0.148
**Anxiety**			
Baseline (20- 80)	37.4 [9.8]	38.2 (7.6]	
42-45 postpartum days (16- 64)	21.7 [4.1]	26.1 (6.0]	-4.7 (-9.2 to –0.17), 0.042

Values are presented as mean [standard deviation] unless otherwise indicated.

a Using the Edinburgh Postnatal Depression Scale (EPDS); higher scores indicate the more severe symptoms

b Using the State scale of the State-Trait Anxiety Inventory (STAI) to assess baseline anxiety, and the Postpartum Specific Anxiety Scale–Research Short-Form (PSAS-IR-RSF) to assess anxiety at 42-45 days postpartum; higher scores indicate the more severe symptoms. Because anxiety was measured with different validated tools at the two time points, scores are not directly comparable across time.

*in the total group, n = 60 in each group; in the VLBW subgroup, n=35 in the probiotic group at 42-45 postpartum days due to loss to follow-up after infant death.

†AMD: adjusted mean difference (probiotic minus placebo) at 42-45 days postpartum, estimated using ANCOVA and adjusted for stratification factors (twin status and birth-weight subgroup) and baseline EPDS score for depression and baseline STAI score for anxiety.

*28 in the postpartum follow-up due to one loss to follow-up

[†]The trial primary outcome,

Supplementary Table 3. Neonatal outcomes in infants weighed 1501–2000 g at birth[a]

Neonatal outcomes	Probiotic (n = 28)	Placebo (n = 32)	MD (95% CI)/	P-value
**Total serum bilirubin (mg/dL)***				
Baseline	7.8 [1.5]	8.4 [1.8]		
4th day of intervention	6.0 [1.6]	5.9 [1.7]	0.026 (-0.85 to 0.91) [b]	
7th day of intervention	4.5 [1.4]	4.4 [1.6]		
**Secondary outcomes**				
Phototherapy duration (days, since birth)*	3 [2, 5]	4 [2.2, 5]		0.54[c]
Weight gain (g) during 42-45th days*	909 [482]	1013 [440]	137 (-163 to 436)[d]	
Infant nutrition				
Duration of total parenteral nutrition (days, since birth)	0 [0, 0]	0 [0, 0]		0.38[c]
Time to achieve full enteral feeding (days, since birth)	10 [8.2, 14.5]	9.5 [7.2, 12.5]		0.40[c]
Breast milk received during the initial 7 days of the intervention (mL)	348 [296, 396]	352 [180, 512]		0.59[c]
Length of hospital stay (days of birth)	12 [10, 19.7]	11 [9, 15]		0.62[c]
Duration continuous positive airway pressure (days, since birth)	2 [1, 4]	1.5 [1, 2.7]		0.44[c]
			**RR (95% CI)**	
Any serious problems*†	1 (3.6)	2 (6.5)		> 0.99[e]
Death	0 (0)	1 (3.1)		
Necrotizing enterocolitis	0 (0)	0 (0)		
Positive blood culture	0 (0)	0 (0)		
Bronchopulmonary dysplasia	1 (3.6)	0 (0)		
Retinopathy of prematurity	0 (0)	1 (3.2)		
Intraventricular hemorrhage (grade II-IV)	0 (0)	0 (0)		
Received blood transfusion	6 (21.4)	2 (6.5)		0.134[e]
Received phototherapy	28 (100)	31 (96.9)		> 0.99[e]
Feeding intolerance on the 7th day of intervention	0 (0)	2 (6.3)		0.49[e]
Nutrition with only breast milk at the discharge	22 (78.6)	24 (75.0)	1.11 (0.56 to 2.19)[f]	
Hospital re-admission	1 (3.6)	2 (6.5)		> 0.99[e]

Values are presented as number (%), mean [SD], or median [Q1, Q3], unless otherwise indicated.

* Total serum bilirubin was primary outcome and the other designated outcomes were main secondary outcomes in the trial. The others were additional secondary outcomes.

†Serious problems (composite outcome) included: death, necrotizing enterocolitis, positive blood culture, bronchopulmonary dysplasia, intraventricular hemorrhage (grade two or over), or retinopathy of prematurity

[a] Subgroup results are exploratory and underpowered.

[b]Repeated Measures ANOVA was performed, with the values at day 4 and day 7 as dependent variables, the baseline value as covariate, and the study group and stratification factors (twin status and birth-weight subgroup) as between-subject factor, with Sidak adjustment for multiple comparisons; Neither the group-by-time interaction nor the post-intervention time-specific effects were significant (p = 0.63 and 0.83).

[c]Mann-Whitney test

[d]ANCOVA adjusted for birth weight and stratification factors (twin status and birth-weight subgroup)

[e]Fisher's Exact test

[f]Chi-square test
